# Longitudinal gut microbiota composition of South African and Nigerian infants in relation to tetanus vaccine responses

**DOI:** 10.21203/rs.3.rs-3112263/v1

**Published:** 2023-07-03

**Authors:** Saori C. Iwase, Heather B. Jaspan, Anna-Ursula Happel, Susan P. Holmes, Alash’le Abimiku, Sophia Osawe, Clive M. Gray, Jonathan M. Blackburn

**Affiliations:** University of Cape Town; Seattle Children’s Hospital; University of Cape Town; Stanford University; University of Maryland, Baltimore County; Institute of Human Virology; Stellenbosch University; University of Cape Town

**Keywords:** HIV-exposed uninfected infants, South Africa, Nigeria, gut microbiome, Tetanus toxoid, Vaccine response

## Abstract

**Introduction:**

Infants who are exposed to HIV but uninfected (iHEU) have higher risk of infectious morbidity than infants who are HIV-unexposed and uninfected (iHUU), possibly due to altered immunity. As infant gut microbiota may influence immune development, we evaluated the effects of HIV exposure on infant gut microbiota and its association with tetanus toxoid (TT) vaccine responses.

**Methods:**

We evaluated gut microbiota by 16S rRNA gene sequencing in 278 South African and Nigerian infants during the first and at 15 weeks of life and measured antibodies against TT vaccine by enzyme-linked immunosorbent assay (ELISA) at matched time points.

**Results:**

Infant gut microbiota and its succession were more strongly influenced by geographical location and age than by HIV exposure. Microbiota of Nigerian infants drastically changed over 15 weeks, becoming dominated by *Bifidobacterium longum* subspecies *infantis*. This change was not observed among EBF South African infants. Lasso regression suggested that HIV exposure and gut microbiota were independently associated with TT vaccine responses at week 15, and that high passive antibody levels may mitigate these effects.

**Conclusion:**

In two African cohorts, HIV exposure minimally altered the infant gut microbiota compared to age and country, but both specific gut microbes and HIV exposure independently predicted humoral vaccine responses.

## Background

The mutualistic relationship between microbes and humans begins in early life. Emerging evidence suggests that the colonization of microbes in the gut facilitates the development of the immune system and growth trajectories [1, 2]. Due to the successful prevention of vertical transmission programs, the number of HIV-exposed yet uninfected infants (iHEU) infants has been increasing, particularly in sub-Saharan Africa [3]. Compared to infants who are HIV-unexposed and uninfected (iHUU), iHEU are at higher risk of morbidity and mortality, predominantly due to infectious diseases [4]. This is thought to be linked to their altered immunity [5, 6], which may be secondary to altered gut microbiota. To our knowledge, there are limited longitudinal studies comparing gut microbiota between iHEU and iHUU, and most of them are conducted in a single country [7–10]. Some studies have found few differences [8, 10, 11], whereas clear differences in microbiota profile were observed in Haitian [7] and Nigerian iHEU [9]. Thus far, only one cross-sectional study compared the gut microbiota between iHEU and iHUU in multiple countries, including Belgium, Canada, and South Africa, and suggested that the difference in microbiota by HIV exposure status may be population-specific [12]. Therefore, the effect of geography and HIV exposure on gut microbiota requires further investigation.

Vaccines are critical for protecting infants from infectious diseases and consequent morbidity and mortality. However, multiple factors can influence vaccine immunogenicity, including genetics, nutritional status, and pre-existing immunity [13]. In addition, emerging evidence points to a possible role of the gut microbiome in influencing vaccine response [14]. In Bangladeshi infants, CD4 + T-cell proliferation and IgG against tetanus toxoid (TT) vaccination were positively associated with abundance of Actinobacteria, particularly *Bifidobacterium longum*, until at least 2 years of age [15, 16]. Conversely, vaccine-induced CD4 + T-cell proliferation against TT was negatively associated with the abundance of *Enterobacteriales* and *Pseudomonadales* [15].

To evaluate the contribution of gut microbiota to observed differences in immunity between iHEU and iHUU, we longitudinally compared the gut microbiota of South African and Nigerian infants exposed and unexposed to HIV, and correlated these with TT vaccine responses.

## Methods

### Study Participants

Mothers with and without HIV and their neonates were recruited into a multicentre longitudinal study between September 2013 and November 2017 [17]. Mother-infant pairs were enrolled during the first week post-delivery at the Khayelitsha Site B Midwife Obstetric Unit in Cape Town, South Africa, and the Plateau State Specialist Hospital in Jos, Nigeria. Clinical and demographic data and samples (including stool and blood) were collected. All mothers with HIV received antiretroviral therapy according to local guidelines, and their infants were confirmed as HIV negative by polymerase chain reaction (PCR) at birth and later time points [18, 19]. In addition, iHEU received antiretroviral post-exposure prophylaxis (PEP) after birth and cotrimoxazole starting at 6 weeks of age as per country-specific guidelines [18, 20]. Exclusive breastfeeding was advised to all mothers from delivery for 6 months. Feeding data was collected using a structured questionnaire validated in similar settings [21]. Feeding practices were categorized as “exclusive breastfeeding”, defined as receiving only breastmilk or prescribed medicines since birth, or “mixed feeding”, defined as receiving breastmilk supplemented with other liquids or food or receiving formula. In this analysis, we included stool and plasma collected from term infants during the first and at 15 weeks of life born to mothers without complications during pregnancy or delivery.

### Immunization

Routine childhood vaccinations were given to all infants according to the World Health Organization (WHO) Expanded Program on Immunization [22]. In both countries, infants were vaccinated against TT at 6, 10, and 14 weeks. South African infants received DTaP (Diptheria-Tetanus-acellular Pertussis), while Nigerian infants received DTwP (Diptheria-Tetanus-Whole-cell-Pertussis). Pregnant mothers were given booster TT vaccination in Nigeria.

### Sample collection, DNA extraction and 16S rRNA gene sequencing

Faecal samples were collected from diapers, avoiding the surface. Samples were placed on ice immediately, transferred to the lab within 6 hours, and stored at −40 to −20°C until analysis. Samples were thawed and treated with a cocktail of mutanolysin (300 U/ml, Sigma Aldrich), lysozyme (45,000 U/ml, Sigma Aldrich), and lysostaphin (24 U/ml, Sigma Aldrich) in 300μl PBS for 1 hour at 37°C. Samples were then mechanically disrupted by bead-beating at 50 Hz for 10 min using the Qiagen TissueLyser LT [23]. DNA was extracted using the PowerSoil DNA extraction kit (Qiagen), following the manufacturer’s protocol. For cross-contamination filtering, genomic DNA was extracted from mock bacterial community cells with equal colony-forming units from each of the 22 known species (HM-280, BEI). Extracted genomic DNA was subjected to PCR amplification in triplicates using primers targeting the V3-V4 region (357F/806R primers) of the 16S rRNA gene, as described previously [24]. Negative controls for DNA extraction and PCR were included. Amplified libraries were purified using AMPure XP beads (Beckman Coulter), quantitated using Quant-iT dsDNA High Sensitivity Assay Kits (ThermoFisher), pooled in equal molar amounts, and paired-end sequenced using a MiSeq Reagent Kit V3 (600-cycle, Illumina). Following demultiplexing, barcode primers were removed using Cutadapt (version 3.4) [25], and reads were processed using DADA2 (version 1.19.2) [26] within the R framework (R version 4.0.4) [27]. Taxonomic classification of amplicon sequence variants (ASVs) was done using an updated SILVA training set (version 132) [28], available at https://github.com/itsmisterbrown/updated_16S_dbs [29]. Contaminant ASVs were identified and removed using the decontam package (version 1.16.0) [30]. Samples with less than 2,000 filtered reads were excluded from the downstream analysis.

### Plasma IgG anti-TT antibodies

Human IgG antibodies against TT in plasma were measured by enzyme-linked immunosorbent assay (ELISA) following the manufacturer’s protocol (TECAN, IBL International GmbH) on samples obtained from infants at 1 and 15 weeks and a subset of mothers from the Nigerian site at 1 week postpartum. The optical density at 450 nm was measured by an ELISA microplate reader (BioTek ELx808 absorbance plate reader), and a standard curve was generated using the readings from the calibrators included on each plate and used to calculate the individual titers (IU/ml). Samples on each plate were run in duplicate, and the average of both titers was used as the final titer. Previously tested positive samples were incorporated into subsequent runs as in-house controls.

### Data analysis

Differences in study cohort characteristics were assessed using the Student’s t-test (parametric continuous variables), Wilcoxon signed-rank test (non-parametric continuous variables), and Chi-squared test (parametric categorical variables). Spearman’s rank correlation coefficient (R) was used to analyse associations between groups. Bacterial community analysis was done using the phyloseq (version 1.40.0) [31] and vegan (version 2.4.6) [32] packages. The ASV table was normalized (i.e., transformed to relative abundance * median sample read depth) and filtered so that each ASV had at least 10 counts in at least 20% of the samples or had a total relative abundance of at least 0.1%. Shannon index was calculated as a measure of α-diversity. Comparison of microbial community composition between groups was evaluated by principal coordinate analysis (PCoA) and permutational multivariate analysis of variance (PERMANOVA) using the adonis2 function in the vegan package [32], based on the Bray-Curtis dissimilarity and 999 permutations. Partitioning around medoids (PAM) clustering was applied to determine the optimal *k* using the cluster package (version 2.1.4) [33]. Analysis of Compositions of Microbiomes with Bias Correction (ANCOM-BC; version 1.6.4) [34] was used to identify significantly differentially abundant ASVs through pair-wise comparisons with an adjusted p-value of < 0.05, and loge fold change of > 0.5 or < −0.5. P-values of anti-TT IgG titers were compared by Wilcoxon signed-rank tests and adjusted for multiple comparisons using the Benjamini–Hochberg method. To identify factors associated with infant anti-TT IgG titers at 15 weeks of age, we applied a Lasso regression using the glmnet package (version 4.1.4) [35]. Since microbiome compositional data is often highly skewed, we employed rank-based transformation [36] for the regression analysis using the top 50 ASVs among infants who had microbiota data available at both week 1 and week 15. After the transformation, the most abundant bacterial taxon within the sample was given the highest score of 50, and the least abundant bacterial taxon was given a score of 1. The rank-transformed ASVs, infant anti-TT IgG titers at 1 week of age (indicative of passive maternal antibody transfer), and HIV exposure status were used as explanatory variables. Models were created according to the infant’s age and geographical location separately. The predictive models were validated by 10-fold cross-validation using cv.glmnet() function in the glmnet package [35]. Lambda value that gave the lowest model error was used as a tuning parameter. Variables that fitted within the regression model were considered to be predictor variables for the TT vaccine response. P-values < 0.05 and 95% confidence intervals were used to assess statistical significance.

## Results

### Cohort characteristics

Overall, there were 278 mother-infant pairs included in this analysis; 82 were from South Africa, and 196 were Nigerian. Several demographic and socioeconomic characteristics differed by study site (Table 1). At enrolment, Nigerian mothers were older (mean age 31 (standard deviation (SD) ± 5.31) versus 28 (SD ± 5.38) years; *P* = 0.001) with higher gravidity (median 2 [interquartile range (IQR) 1–4] versus 1 [IQR 1–2]; *P* < 0.001) and lower body weight (mean 62.87 (SD ± 11.51) versus 72.69 (SD ± 13.86) kg; *P* < 0.001) than South African mothers. While electricity was equally available for participants from both countries, significantly more mothers in South Africa had a refrigerator and running water at home, and significantly more Nigeria mothers lived in formal housing (all *P* < 0.001). The weight-for-length z score (wflz) of Nigerian infants was significantly lower than that of South African infants at 15 weeks of age (0.54 versus 0.86, *P* = 0.023). All Nigerian infants were exclusively breastfed (EBF) until 15 weeks of life, whereas only 58.5% of South African mothers reported still EBF at 15 weeks postpartum (*P* < 0.001). There were no significant differences in cohort characteristics by HIV exposure, except mothers of iHUU had higher formal education than mothers of iHEU (*P* = 0.002; Supplementary Table S1).

### Gut microbiota differs substantially between South African and Nigerian infants in the first week of life

**Figure 1:**
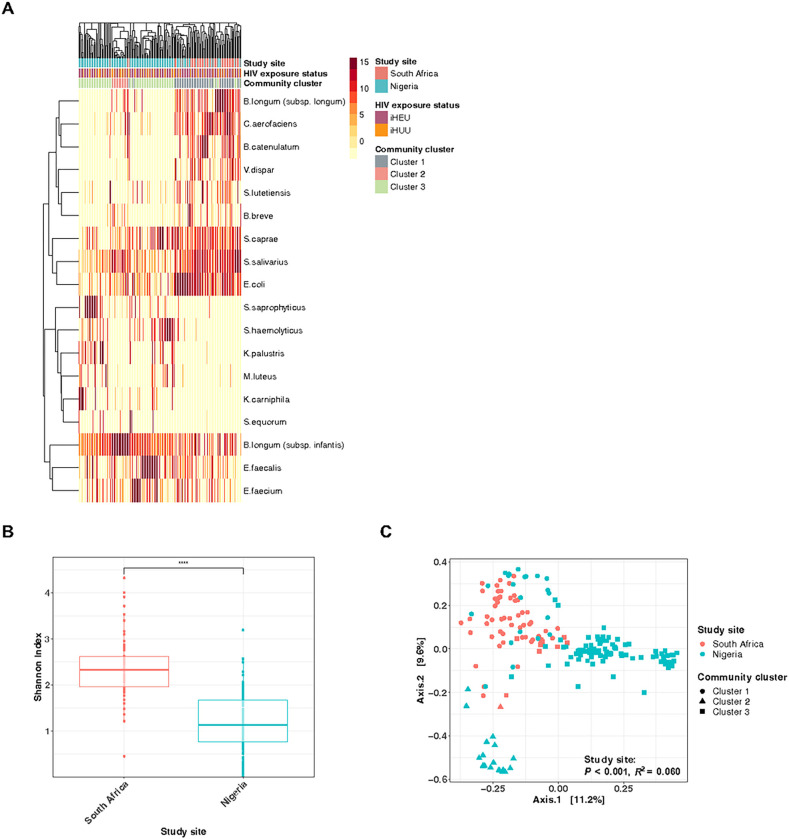
Geographical location strongly affects gut microbiota among African infants in the first week of life. (A) Heatmap of the top 20 taxa in the gut microbiota of South African and Nigerian infants in the first week of age. Study site, HIV exposure status, and community cluster types (based on PAM clustering; *k* = 3) were annotated. Bacterial taxa were merged and annotated at the species level. (B) Comparison of α-diversity (Shannon index) between South African and Nigerian infants during the first week of life. (C) PCoA and PERMANOVA test (Bray-Curtis dissimilarity) of gut microbiota during the first week of age, coloured by study site and shaped by community groups, based on PAM clustering (*k* = 3). iHEU, infants who are HIV-exposed uninfected; iHUU, infants who are HIV-unexposed uninfected; PCoA, principal coordinate analysis; PERMANOVA, permutational multivariate analysis of variance; PAM, partitioning around medoids. **** *P* < 0.0001.

Of the 524 samples sequenced, 442 samples passed the quality filtering. Of these, 164 (47 South African and 117 Nigerian) out of the 278 infants had gut microbiota data available at both time points. Gut microbiota composition differed significantly by study site during the first week of life (Fig. 1A). Within-sample microbial diversity (Shannon index) was higher among South African than Nigerian infants (*P* < 0.0001; Fig. 1B). In addition, microbial community composition was significantly different by geographical location, although site only explained 6% of the community composition (Fig. 1C; PERMANOVA *P* < 0.001). Geographical location remained significantly associated with α- and β- diversity when adjusted for sequencing batch or demographic factors that significantly differed between countries, namely maternal marital status, weight, age, gravity, education level, occupation, type of house, access to a refrigerator or running water, mode of delivery and infant gestational age (*P* < 0.001 for both α- and β- diversity). In addition, α- and β- diversity remained significantly different by the geographic location when the comparison was made strictly among samples collected in the first day of life (Supplementary Figure S1).

At baseline, most South African infants had gut microbiota consisting of (1) Actinobacteriota, including several *Bifidobacterium* species (such as *B. longum* subspecies *longum, B. catenulatum*, and *B. breve*) and *Collinsella aerofaciens*, (2) Firmicutes, including *Streptococcus* species (such as *S. salivarius*, *S. caprae*, and *S. lutetiensis*) and *Veillonella dispar* and (3) Proteobacteria which mainly consist of *E. coli*, which was named “cluster 1” identified by PAM clustering (Fig. 1A). On the other hand, the majority of Nigerian infants’ gut microbiota was classified as community cluster 3, dominated by (1) Actinobacteriota, mainly *B. longum* subspecies infantis and (2) Firmicutes, including *Staphylococcus* species (such as *S. haemolyticus* and *S. saprophyticus*) and *Enterococcus* species (such as *E. faecalis* and *E. faecium*).

### Age is a major driver of microbiota development, but microbial succession differs between sites

**Figure 2:**
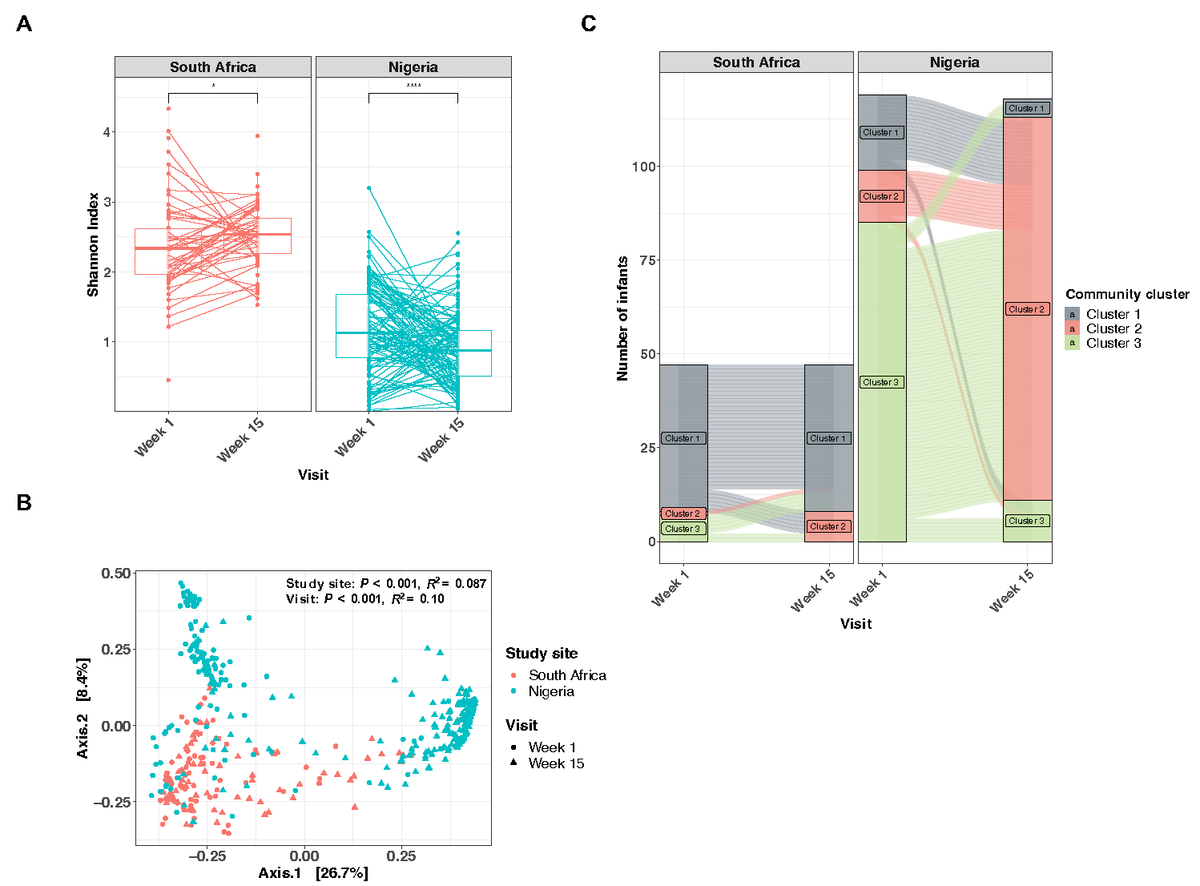
Longitudinal transition of gut microbiota is distinct among infants in South Africa and Nigeria. (A) The transition of α-diversity (Shannon index) of infant gut microbiota over the first 15 weeks of age in South Africa and Nigeria. (B) PCoA and PERMANOVA test (Bray-Curtis dissimilarity) of gut microbiota at 1 week and 15 weeks of age, coloured by study site and shaped by visit. (C) Alluvial plot showing the transition of cluster groups from week 1 to week 15 at each study site. Samples were grouped according to the PAM clustering (*k* = 3), indicated by colour. PCoA, principal coordinate analysis; PERMANOVA, permutational multivariate analysis of variance; PAM, partitioning around medoids. * *P*< 0.05; **** *P* < 0.0001. *B. longum* = *B. longum* subspecies*infantis.*

**Figure 3:**
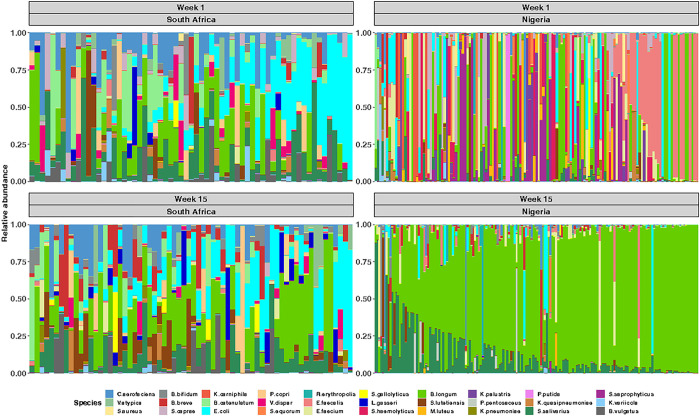
Infants’ gut microbial succession over the first 15 weeks differs substantially between the study sites. Relative abundance plot of most abundant 30 taxa of South African and Nigerian infants at the 1 week and 15 weeks of age. Each column represents individual participants.

We next assessed the gut microbiota longitudinally. The α-diversity in South African infants increased significantly from week 1 to week 15 (*P* = 0.036), while α-diversity in Nigerian infants significantly decreased (*P* < 0.0001) (Fig. 2A). There was a clear separation of microbial community composition among Nigerian samples by age, which was less evident for South African infants (Fig. 2B). In agreement, the dominant bacterial changed only marginally from week 1 to week 15 among South African infants, while Nigerian infants experienced a shift from a Firmicutes-dominated microbiota (cluster 3) to one dominated by *Bifidobacterium infantis* and *Streptococcus salivarius* (cluster 2) at 15 weeks of age (Fig. 2C; Fig. 3). The significant differences observed between countries in α- and β-diversity remained the same over the 15 weeks when the comparison was strictly among EBF infants or vaginally delivered infants (Supplementary Figure S2–3).

### HIV exposure has a subtle effect on the gut microbiota regardless of the geographical location

There were no significant differences in α-diversity (Supplementary Figure S4A), β-diversity (Supplementary Figure S4B), or PAM cluster transition (Supplementary Figure S4C-D) by HIV exposure status in either country. Differential abundance testing using ANCOM-BC analysis of differential abundance in microbiome data was performed adjusting for feeding mode at the week 15 time point [34]. Several bacterial taxa significantly associated with HIV exposure status in South Africa (Table 2). Several *Enterococcus* species (*E. faecalis, E. faecium, E. gilvus*, and *E. raffinosus*) were significantly more abundant in iHEU than iHUU at week 15 (Loge fold change (LFC): 0.61, 0.57, 1.02, and 0.76, respectively). Moreover, *Collinsella aerofaciens* (LFC: 0.72 at week 1 and 1.18 at week 15) and *Klebsiella quasipneumoniae* (LFC: 0.84 at both week 1 and week 15), which are known to be pathobionts [37, 38], were consistently more abundant in iHEU during the first 15 weeks of life. In contrast, no bacterial taxa were differentially abundant by HIV exposure in the Nigerian cohort.

### Maternal HIV status and infant gut microbes influence infant TT vaccine response

**Figure 4:**
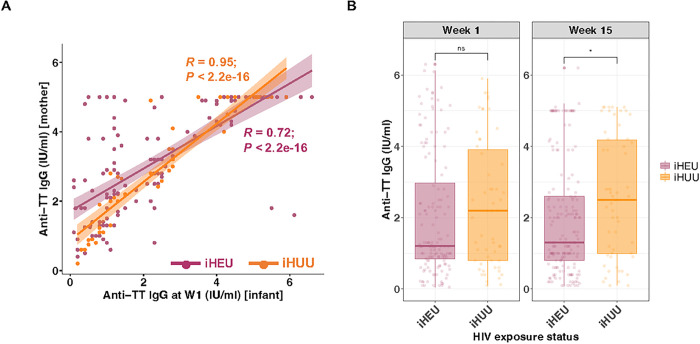
Passive maternal antibody and HIV exposure are both associated with infant TT response. (A) Scatter plot and Spearman’s rank correlation coefficients (R) of anti-TT IgG titers (IU/ml) between Nigerian mothers (y-axis) and their infants at week 1 (x-axis). Dots and lines of best fit were coloured by HIV exposure status. (B) Comparison of anti-TT IgG titers between iHEU and iHUU at week 1 and week 15. P-values comparing anti-TT IgG titers were adjusted for multiple comparisons using Benjamini–Hochberg method. W1, 1 week of age; iHEU, infants who are HIV-exposed uninfected; iHUU, infants who are HIV-unexposed uninfected; TT, tetanus toxoid; ns, not significant. * *P* < 0.05.

In Nigeria, it is recommended that pregnant women receive TT booster vaccinations, whereas this is not policy in the Western Cape, South Africa [39]. Therefore, not surprisingly, infant anti-TT IgG titers in the first week of life, representing maternally transferred antibodies, were significantly lower among South African infants than Nigerian infants (median 1.0 versus 1.5 IU/ml, adj *P* = 0.002; Supplementary Figure S5A). In contrast, titers did not differ between South African and Nigerian infants at 15 weeks of age (median 1.9 versus 1.6 IU/ml, adj *P* = 0.280). We investigated the correlation of TT vaccine response between mother and infant pairs living in Nigeria. Anti-TT IgG titers were strongly correlated at week 1. However, iHEU mother-infant anti-TT IgG titers showed a lower Pearson’s correlation coefficient compared to iHUU (R: 0.72 versus 0.95) (Fig. 4A). The correlation between maternal and infant anti-TT IgG titers was no longer evident by 15 weeks of age in either iHEU and iHUU (Supplementary Figure S5B). We did not see any difference in anti-TT IgG titers among mothers by their HIV status (Supplementary Figure S5C). However, iHEU had significantly lower TT IgG concentrations than iHUU at 15 weeks of life (*P* = 0.016), and this remained significant after adjusting for multiple comparisons (adj *P* = 0.031; Fig. 4B). However, the difference between iHEU and iHUU at week 15 was no longer statistically significant when infants were compared separately by study site (adj *P* = 0.290 in South Africa and adj P = 0.180 in Nigeria; Supplementary Figure S5D).

**Figure 5:**
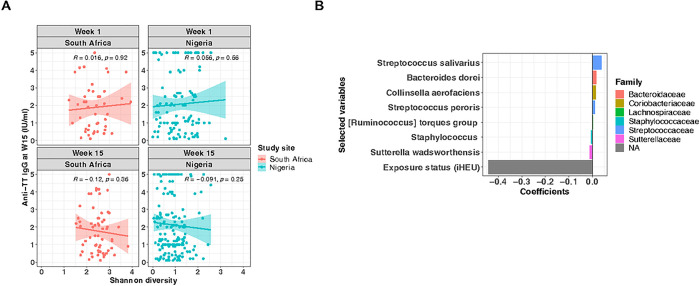
HIV exposure status and gut microbiota are independently associated with TT vaccine response. **(A)** Correlation analysis of infants’ anti-TT IgG titers (IU/ml) measured at 15 weeks of age and α-diversity (Shannon index) at each study site and visit. Spearman’s rank correlation coefficients (R) were indicated on each panel. (B) Rank-transformed top 50 ASVs (at either week 1 or week 15), HIV exposure status, and anti-TT IgG titer data at week 1 were used as explanatory variables for the Lasso regression to assess the association with TT vaccine response at 15 weeks of age. Each model was constructed separately based on geographical location and time point. The optimal coefficient tuning parameter (lambda.min) was chosen using 10-fold cross-validation. Selected variables and their glmnet coefficients were plotted. Color of the bars represents taxonomy at the family level. Week 1 ASVs and HIV exposure status were associated with week 15 TT vaccine response among South African infants. No variables were selected for the Nigerian cohort. TT, tetanus toxoid; W15, 15 weeks of age; iHEU, infants who are HIV-exposed uninfected; ASVs, amplicon sequence variants.

Since gut microbiome is thought to modulate the development of the immune system [15], we intended to investigate the relationship between infant gut microbiota and TT vaccine response at week 15. We did not see consistent correlations between 15-week anti-TT IgG titers and Shannon diversity of either time point (Fig. 5A).

To further explore factors associated with infant TT vaccine response at week 15, we conducted Lasso regression analysis. Rank-transformed top 50 ASVs at either week 1 or week 15, HIV exposure status, and anti-TT IgG titers at week 1 were included as explanatory variables to investigate the predictor, TT vaccine response at 15 weeks of age. In South Africa, infant HIV exposure status showed a strong negative association with 15-week TT vaccine response (β-coefficient = −0.44), and the rank-transformed bacterial taxon abundance at week 1, including *Streptococcus salivarius* (β-coefficient = 0.038), *Bacteroides dorei* (β-coefficient = 0.016), *Collinsella aerofaciens* (β-coefficient = 0.015), and *Sutterella wadsworthensis* (β-coefficient = −0.011) were independently associated with the vaccine response, albeit with weaker β-coefficients than HIV-exposure (Fig. 5B; Supplementary Table S2). In contrast, no variables were selected as predictors of the TT vaccine response in the Nigerian cohort. Previously, it has been shown that passively transferred maternal antibody interferes with infant TT vaccination response [40]. Since Nigerian infants showed significantly higher maternal antibodies than South African infants at week 1 (Supplementary Figure S5A), we speculated that these maternal TT antibodies may have masked any associations underlying the infant TT vaccine response. For this reason, we re-assessed the Lasso regression without including week 1 anti-TT IgG data in the explanatory variables (Supplementary Figure S6; Supplementary Table S3). Although there was no change in the result for the South African infants (Supplementary Figure S6A), HIV exposure and several bacteria present at 15 weeks of age, including *S. salivarius*, were independently associated with the TT vaccine response in Nigerian infants (Supplementary Figure S6B). However, the β-coefficients for all selected predictors were small, including HIV exposure status.

## Discussion

This is one of the largest studies that longitudinally compared the gut microbiota between iHEU and iHUU in two settings and investigated the association with their vaccine responses. Our findings suggest that the country of origin was the most influential factor in the infants’ gut microbiota at week 1, which also strongly affected its succession over the first 15 weeks of life. Both feeding and delivery modes have been shown to influence infant gut microbiota [41]. Since these demographic characteristics significantly differed between our South African and Nigerian cohorts, we explored their potential effects on the infant gut microbiome. Notably, a comparison restricted to EBF infants showed that α- and β-diversity at week 15 remained significantly different between the countries. Similarly, the difference was independent of mode of delivery. In addition, analysis of stool samples collected shortly after birth suggested that the difference in gut microbiota profile was already prominent before feeding was established. Collectively, these data indicate that geographical location strongly influences the initial seeding of gut microbes, and this affects the trajectory of microbiota regardless of feeding practices [42]. Notably, the term “geography” includes not only the physical location (rural versus urban) but also extends to socioeconomics, genetics, diet, climate, and ethnicity [42, 43].

Microbiota among Nigerian infants transitioned drastically over the first 15 weeks of life, such that at 15 weeks, *B. infantis* was the dominant taxon, with some *S. salivarius*. Both are commonly found in breast milk and gut microbiota among breastfed infants [44]. *Bifidobacteria* benefit human health and are often used as probiotics [45]. Moreover, *Streptococcus salivarius*, classified as a lactic acid bacterium (LAB), has been shown to have probiotic properties [46]. However, the drastic changes in microbiota over the 15 weeks only occurred among Nigerian infants, not South African infants. Plausible explanations for this may be the difference in profiles of maternal breastmilk microbiota and human milk oligosaccharides (HMOs) influenced by genetics, ethnicity, diet, and body mass index (BMI) [47]. For instance, a higher maternal BMI is associated with reduced *Bifidobacterium* in breastmilk [48], which might suggest that South African mothers, who had higher mean weight, had less *Bifidobacterium* in their breastmilk than Nigerian mothers, leading to less *Bifidobacterium* in their infants’ gut. An additional explanation is that Nigerian mothers in this setting have a diet rich in fermented foods, whereas South African mothers may have a more Westernised diet [49, 50]. South African infants also had higher relative abundance of *B. longum* but lower relative abundance of *B. infantis* at baseline.

Nigerian infants had significantly higher anti-TT IgG titers, likely due to maternal immunisation and consequent high passive maternal antibody transfer at week 1 compared to South African infants [51]. In contrast, in the Western Cape region where our South African cohort was recruited, there was no routine TT booster vaccination for pregnant women due to the prolonged absence of neonatal TT cases in the province [39]. Notably, anti-TT IgG levels post-vaccination among Nigerian infants remained the same as at week 1 and were comparable with South African infants. This inferior induction of TT titers observed in Nigerian infants may be explained by the inhibition of TT vaccine response by passively transferred high maternal antibodies, as previously described [40].

Lasso regression models also suggested that *in-utero* HIV exposure and relative abundance of several bacterial taxa at week 1 were independently associated with later TT vaccine response in South Africa but not Nigeria. The higher passive antibody levels observed in Nigerian infants may have mitigated the effects of HIV exposure and microbiota on the infant vaccine response. In fact, excluding the week 1 titer data from the regression model indicated that the HIV exposure and several microbes found at 15 weeks of age were independently associated with the infant TT vaccine response among Nigerian infants. In line with our assumption, removing week 1 titer data from the regression model did not change the Lasso regression result in South African infants, who showed much lower passive maternal antibody transfer. Interestingly, *S. salivarius* relative abundance was predictive of improved TT titers in both cohorts. Since the microbiota at week 1 among South African infants was associated with TT vaccine response at week 15, this suggests that in some settings, vaccine responses could potentially be modified using an early-life microbiome intervention where maternal vaccination is not possible.

## Conclusions

This study showed that the transition of infant gut microbiota was strongly dependent on geographical location and age, while effect of *in-utero* HIV exposure was modest. However, maternal HIV status was negatively associated with the passive maternal anti-TT antibody transfer, and the negative effect of HIV exposure on TT vaccine response persisted over the first 15 weeks of life among iHEU. In addition, there were independent associations of specific gut microbes and HIV exposure with infant humoral response to TT at 15 weeks of age.

## Figures and Tables

**Table 1:** Cohort characteristics by Site

		South Africa	Nigeria	*P*
		(N = 82)	(N = 196)	
**Maternal characteristics**				
**Mother’s age at delivery (years; mean (SD))**		28 (5.38)	31 (5.31)	0.001
**Education (n; %)**	None	0 (0.0)	2 (1.0)	< 0.001
	Elementary	5 (6.1)	65 (33.2)	
	Secondary	72 (87.8)	73 (37.2)	
	Higher	5 (6.1)	56 (28.6)	
**Unemployed (n; %)**		59 (72.0)	6 (3.1)	< 0.001
**Formal housing (n; %)**		33 (40.2)	185 (94.4)	< 0.001
**Electricity (n; %)**		78 (95.1)	178 (90.8)	0.332
**Refrigerator (n; %)**		70 (85.4)	96 (49.0)	< 0.001
**Running water (n; %)**		38 (46.3)	48 (24.5)	0.001
**Marital status (n; %)**	Married/ living together	25 (30.5)	186 (94.9)	< 0.001
	Single	57 (69.5)	10 (5.1)	
**Gravidity (n; median [IQR])**		1 [1, 2]	2 [1, 4]	< 0.001
**Mother’s weight at enrollment (kg; mean (SD))** ^a^		72.69 (13.86)	62.87 (11.51)	< 0.001
**Infant characteristics**				
**iHEU (n; %)**		61 (74.4)	141 (71.9)	0.787
**Male (n; %)**		41 (50.0)	94 (48.0)	0.858
**Gestational age at delivery (weeks; median [IQR])**		39.30 [38.02, 40.38]	39.95 [38.98, 40.62]	0.011
**Vaginal delivery (n; %)**		82 (100.0)	167 (85.2)	0.001
**Wflz at W15 (median [IQR])** ^b^		0.86 [0.32, 1.90]	0.54 [−0.64, 1.42]	0.023
**Mode of feeding at W15 (n; %)**	Exclusive breastfeeding	48 (58.5)	196 (100.0)	< 0.001
	Mixed feeding	34 (41.5)	0 (0.0)	

IQR, Interquartile range; SD, Standard deviation; iHEU, infants who are HIV-exposed uninfected; W15, 15 weeks of age; Wflz, Weight-for-length z score.

a Missing data from 5 Nigerian participants

b Missing data from 41 participants (South Africa, n = 16; Nigeria, n = 25).

**Table 2:** ANCOM-BC analysis of gut microbiota in South African HIV-exposed versus un-exposed infants.

Taxonomy (Genus, Species)	Taxon ID	LFC^a^
**At 1 week of age**		
*Klebsiella variicola*	ASV46	1.22
*Sutterella* (unclassified)	ASV150	1.02
*Holdemanella* (unclassified)	ASV53	1.00
*Parabacteroides merdae*	ASV101	0.98
*Catenibacterium* (unclassified)	ASV218	0.96
*Blautia obeum*	ASV59	0.93
*Senegalimassilia* (unclassified)	ASV145	0.87
*Bifidobacterium breve*	ASV10	0.84
*Klebsiella quasipneumoniae*	ASV36	0.84
*Libanicoccus* (unclassified)	ASV153	0.81
*Blautia* (unclassified)	ASV225	0.80
*Ruminococcus torques* group (unclassified)	ASV75	0.72
*Collinsella aerofaciens*	ASV25	0.72
*Subdoligranulum* (unclassified)	ASV251	0.70
*Bacteroides vulgatus*	ASV83	0.70
*Sutterella* (unclassified)	ASV496	0.67
*Klebsiella pneumoniae*	ASV39	0.66
*Megamonas* (unclassified)	ASV169	0.65
*Romboutsia ilealis*	ASV93	0.64
*Senegalimassilia* (unclassified)	ASV171	0.64
*Faecalibacterium* (unclassified)	ASV505	0.58
*Fusobacterium mortiferum*	ASV278	0.57
*Enterococcus faecium*	ASV7	0.57
*Parabacteroides distasonis*	ASV138	0.54
*Actinomyces* (unclassified)	ASV668	−0.54
*Parabacteroides distasonis*	ASV203	−0.57
**At 15 weeks of age**		
*Streptococcus gallolyticus*	ASV44	1.33
*Collinsella aerofaciens*	ASV25	1.18
*Clostridium innocuum* group (unclassified)	ASV336	1.13
*Enterococcus gilvus*	ASV157	1.02
*Klebsiella quasipneumoniae*	ASV42	0.84
*Veillonella atypica*	ASV163	0.83
*Enterococcus raffinosus*	ASV338	0.76
*Enterococcus* (unclassified)	ASV40	0.71
*Bifidobacterium adolescentis*	ASV296	0.71
*Enterococcus raffinosus*	ASV51	0.71
*Lactococcus lactis*	ASV206	0.66
*Enterococcus faecalis*	ASV5	0.61
*Granulicatella* (unclassified)	ASV681	0.59
*Dorea formicigenerans*	ASV229	0.59
*Faecalibacterium prausnitzii*	ASV103	0.52
*Staphylococcus* (unclassified)	ASV41	0.51
*Lactobacillus rhamnosus*	ASV191	−0.51
*Klebsiella michiganensis*	ASV266	−0.51
*Lactobacillus gasseri*	ASV161	−0.53
*Prevotella copri*	ASV405	−0.55
*Megasphaera elsdenii*	ASV167	−0.58
*Olsenella* (unclassified)	ASV97	−0.58
*Prevotella* (unclassified)	ASV176	−0.83
*Bacteroides caccae*	ASV552	−0.93
*Olsenella* (unclassified)	ASV120	−1.89
*Ruminococcus torques* group (unclassified)	ASV75	−2.43

a Abundance in iHEU in relation to iHUU.

ANCOM-BC: Analysis of Compositions of Microbiomes with Bias Correction; LFC, Log_e_ fold change; ASV, amplicon sequence variants; iHEU, infants who are HIV-exposed uninfected; iHUU, infants who are HIV-unexposed uninfected.

## Data Availability

Sequencing reads from the 16S rRNA gene profiling are available at the Sequence Read Archive repository hosted by NCBI (accession number PRJNA976299). Code is available upon request.

## Abbreviations

ANCOM-BC: Analysis of Compositions of Microbiomes with Bias Correction
ASVs: Amplicon sequence variants
BMI: Body mass index
DTaP: Diptheria-Tetanus-acellular Pertussis
DTwP: Diptheria-Tetanus-Whole-cell-Pertussis
EBF: Exclusively breastfed
ELISA: Enzyme-linked immunosorbent assay
HMOs: Human milk oligosaccharides
iHEU: Infants who are exposed to HIV but uninfected
iHUU: Infants who are HIV-unexposed and uninfected
IQR: Interquartile range
LAB: Lactic acid bacteria
LFC: Loge fold change
PAM: Partitioning around medoids
PCoA: Principal coordinate analysis
PCR: Polymerase chain reaction
PEP: Antiretroviral post-exposure prophylaxis
PERMANOVA: Permutational multivariate analysis of variance
SD: standard deviation
TT: Tetanus toxoid
Wflz: Weight-for-length z score
WHO: World Health Organization
